# IL-15 Prevents Renal Fibrosis by Inhibiting Collagen Synthesis: A New Pathway in Chronic Kidney Disease?

**DOI:** 10.3390/ijms222111698

**Published:** 2021-10-28

**Authors:** Aurore Devocelle, Lola Lecru, Sophie Ferlicot, Thomas Bessede, Jean-Jacques Candelier, Julien Giron-Michel, Hélène François

**Affiliations:** 1 INSERM UMR-S-MD 1197/Ministry of the Armed Forces, Biomedical Research Institute of the Armed Forces (IRBA), Paul-Brousse Hospital Villejuif and CTSA Clamart, 94807 Villejuif, France; aurore.devocelle@inserm.fr (A.D.); lola.lecru@glpg.com (L.L.); jean-jacques.candelier@universite-paris-saclay.fr (J.-J.C.); 2 Orsay-Vallée Campus, Paris-Saclay University, 91190 Gif-sur-Yvette, France; 3 Service d’Anatomopathologie, Hôpital Bicêtre, AP-HP, 94270 Le Kremlin-Bicêtre, France; sophie.ferlicot@aphp.fr; 4 Service d’Urologie, Hôpital Bicêtre, AP-HP, 94270 Le Kremlin-Bicêtre, France; thomas.bessede@aphp.fr; 5 INSERM UMR_S1155, Tenon Hospital, 75020 Paris, France; 6 Soins Intensifs Néphrologiques et Rein Aigu (SINRA), Hôpital Tenon, AP-HP, Sorbonne University, 75020 Paris, France

**Keywords:** chronic kidney disease, fibrosis, unilateral ureteral obstruction, interleukin-15, myofibroblasts, extracellular matrix

## Abstract

Chronic kidney disease (CKD), secondary to renal fibrogenesis, is a public health burden. The activation of interstitial myofibroblasts and excessive production of extracellular matrix (ECM) proteins are major events leading to end-stage kidney disease. Recently, interleukin-15 (IL-15) has been implicated in fibrosis protection in several organs, with little evidence in the kidney. Since endogenous IL-15 expression decreased in nephrectomized human allografts evolving toward fibrosis and kidneys in the unilateral ureteral obstruction (UUO) model, we explored IL-15’s renoprotective role by pharmologically delivering IL-15 coupled or not with its soluble receptor IL-15Rα. Despite the lack of effects on myofibroblast accumulation, both IL-15 treatments prevented tubulointerstitial fibrosis (TIF) in UUO as characterized by reduced collagen and fibronectin deposition. Moreover, IL-15 treatments inhibited collagen and fibronectin secretion by transforming growth factor-β (TGF-β)-treated primary myofibroblast cultures, demonstrating that the antifibrotic effect of IL-15 in UUO acts, in part, through a direct inhibition of ECM synthesis by myofibroblasts. In addition, IL-15 treatments resulted in decreased expression of monocyte chemoattractant protein 1 (MCP-1) and subsequent macrophage infiltration in UUO. Taken together, our study highlights a major role of IL-15 on myofibroblasts and macrophages, two main effector cells in renal fibrosis, demonstrating that IL-15 may represent a new therapeutic option for CKD.

## 1. Introduction

One of the main challenges in nephrology is the prevention of chronic kidney disease (CKD). CKD is characterized by the development of renal fibrosis, which is the inevitable consequence of continuous accumulation and activation of myofibroblasts, with subsequent extracellular matrix (ECM) deposition in interstitial space leading to organ dysfunction [1,2]. Fibrosis occurs in every type of CKD, regardless of the cause, and it contributes to a progressive and irreversible loss of renal function, which is a process known as end-stage renal disease (ESRD). Thus, targeting myofibroblasts is the cornerstone of antifibrotic therapy owing to the central role of these cells in the production of profibrotic mediators, ECM components, crosslinking enzymes, and inhibitors of matrix metalloproteinases [3]. However, the effective targeting of myofibroblasts in organ fibrosis remains a challenge due to incomplete knowledge regarding their origin and functional contribution. Indeed, the exact origin of the myofibroblasts during CKD is still debated. Contrary to what we initially thought [4], tubular epithelial cells, through epithelial-to-mesenchymal transition (EMT), are not the major source of myofibroblasts as they may also derive from endothelial cells, local fibroblasts, pericytes, and bone marrow-derived fibrocytes [5,6].

The development of fibrosis involves a phase of inflammation, which is characterized by the infiltration of macrophages, lymphocytes, and an increase in circulating cytokines and chemokines, which results in the accumulation of myofibroblasts [7]. Targeting fibrosis remains the main goal to treat CKD; however, most anti-fibrotic therapies such as transforming growth factor-β (TGF-β), a master player in the pathogenesis of renal inflammation and fibrosis [8], have proven disappointing [9,10,11]. Current therapies for CKD still rely mainly on renin–angiotensin system inhibitors and more recently SGLT2 inhibitors [12], but they have limited efficacy and only delay disease progression, highlighting the need for new therapeutic strategies to either stop or reverse fibrosis progression.

Thus, recent advances have been noted in the understanding of particular factors involved in renal fibrogenesis. However, focusing on pathways with available therapies may be a relevant approach. Among the many antifibrotic factors (e.g., hepatocyte growth factor (HGF) [13,14] and bone morphogenetic protein-7 (BMP-7) [14,15], Klotho [16]), the multifunctional innate cytokine interleukin-15 (IL-15) has been implicated in the protection of fibrosis development in the lung [17], pancreas islets [18], and liver fibrosis [19] in animals. In addition, IL-15 is currently tested in renal cancer with a good safety profile [20,21] in humans and may thus represent a safe therapeutic option in human CKD as a repurposing drug. Indeed, IL-15 reverses fibrosis by modulating inflammation by recruiting Foxp3+ Treg cells such as in lung fibrosis but also by reducing the levels of profibrotic cytokines such as TGF-β and by blunting fibroblasts transformation into myofibroblasts [17]. In this context, an older study had shown that IL-15 can counteract TGF-β1-induced myofibroblast differentiation of human fetal lung fibroblasts [22]. Similarly, IL-15 displays a protective role in murine hepatic fibrosis by preserving NK homeostasis and modulating inflammation but also by directly blocking the myofibroblastic differentiation of hepatic stellate cells [19]. Moreover, the pharmacological delivery of IL-15 reverses fibrosis in models of chronic pancreatitis by promoting the induction of INF-γ, producing NK and NKT cells, and reducing TGF-β levels [18]. Therefore, IL-15 may blunt fibrosis by regulating inflammation but also through a direct action on fibroblasts and collagen synthesis. Interestingly, IL-15 may also prevent renal fibrogenesis. Indeed, we and others found that IL-15 counteracts apoptosis and TGF-β induced-EMT in primary tubular epithelial cells through autocrine loops and membrane forms [23,24,25,26]. A renoprotective IL-15 effect was also observed in several in vivo models of acute renal injury through its anti-apoptotic activities [23,24] and through its ability to restrain inflammation by inhibiting monocyte chemoattractant protein 1 (MCP-1) expression [23].

Moreover, it is noteworthy that a decreased expression of endogenous IL-15, concomitantly with an increase of TGF-β, is observed in several chronic inflammatory diseases. This unbalanced IL-15/TGF-β ratio also occurs in animal models with clinical and pathological features of human CKD [23]. In this context, our earlier study has shown that in patients, several nephropathies evolving toward fibrosis, such as acute interstitial nephritis, IgA nephropathy, and diabetic nephropathy, are also characterized by an imbalance of the IL-15/TGF-β ratio with weak intrarenal IL-15 expression and increased TGF-β expression [26].

Therefore, IL-15 has the potential of blunting renal fibrosis, through direct roles in tubule protection and in collagen synthesis by myofibroblasts, but also, indirectly, through the modulation of inflammation. The protective potential of IL-15 in renal fibrosis in vivo has not been evaluated. The present study sought to examine if IL-15 treatment ameliorates tubulo-interstitial fibrosis in a mouse model of unilateral ureteral obstruction (UUO) in which the pathological role of TGF-β is well established and to decipher cellular pathways involved.

## 2. Results

### 2.1. IL-15 Expression Is Reduced in Detransplanted Human Renal Grafts

**Figure 1:**
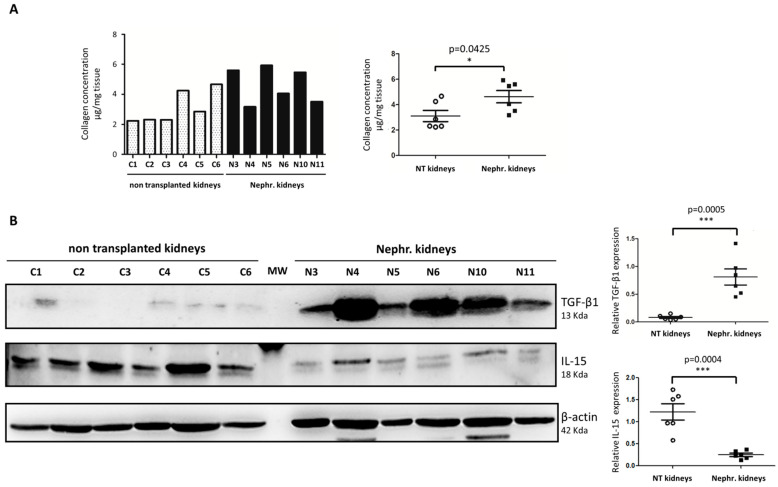
IL-15 expression was reduced in nephrectomized human kidney transplants compared with non-fibrotic “non-transplanted kidneys”. (**A**) Collagen content by hydroxyproline (HYP) assay in well-functioning transplants non-transplanted kidneys (NT kidneys) and nephrectomized allografts (Nephr. kidneys) from patients with renal rejection (4.6 ± 0.4 µg Hyp/mg tissue in Neph. kidneys in comparison to NT kidneys (3.1 ± 0.4 µg Hyp/mg tissue, * *p* < 0.05). (**B**) IL-15 and TGF-β expression were quantified by Western blot. Left, Representative Western blots of one experiment are shown. Right, Bar charts represent IL-15 and TGF-β expression normalized to β-actin. IL-15 (*** *p* < 0.001), TGF-β (*** *p* < 0.001), *n* = 6. Values are means ± SEM.

We first investigated whether IL-15 expression is reduced in nephrectomized allografts from patients with Chronic Allograft Dysfunction and renal fibrosis. To this end, we compared nephrectomized renal grafts with non-fibrotic renal grafts deemed unsuitable for transplantation (because of high cold ischemia time or anatomical reasons but with otherwise good renal function). We first confirmed that fibrotic nephrectomized renal allografts showed higher total collagen content (*p* < 0.05, *n* = 6) by colorimetric quantification of hydroxyproline (Hyp), which is an amino acid specific to collagen (Figure 1A), and increased levels of active TGF-β (*p* < 0.001, *n* = 6) by Western blot (Figure 1B) in comparison with ”non-transplanted (NT) kidneys”(*n* = 6). We next found that IL-15 expression was drastically reduced in nephrectomized allografts compared with “NT kidneys” (*p* < 0.001, *n* = 6) (Figure 1B).

### 2.2. IL-15/IL-15Rα Expression Decreases in the Obstructed Kidneys in UUO Model

**Figure 2:**
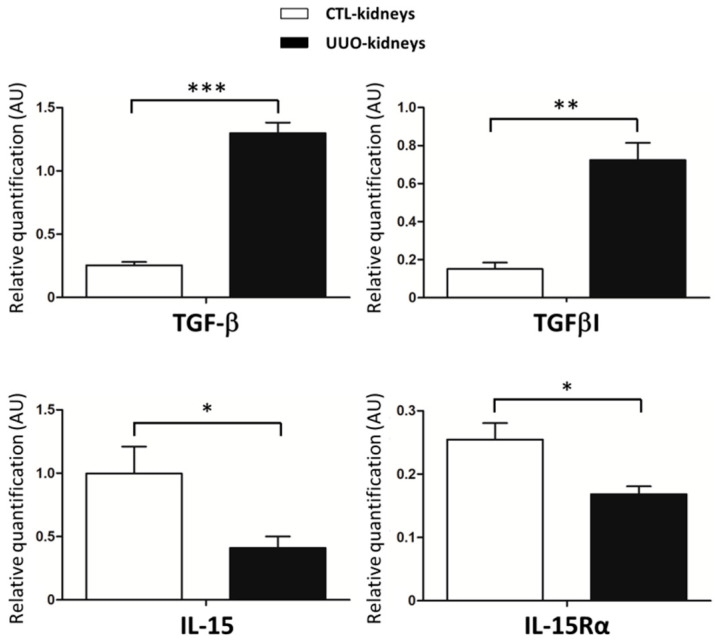
IL-15 expression was reduced in 8 d-post obstructed kidneys. C57BL/6 mice were subjected (black bars) or not (white bars) to UUO; kidneys were removed 8 days after surgery. Quantitative PCR analysis of IL-15, IL-15Rα, TGF-β, and TGF-βI mRNA normalized to Gus B mRNA in obstructed kidneys (UUO-kidneys) and contralateral (CTL-kidneys). AU, arbitrary units. Values are means ± SEM from seven mice by group (two-way ANOVA test, * *p* < 0.05, ** *p* < 0.01, *** *p* < 0.001).

Then, we decided to decipher the possible protective role of IL-15 in a model of renal fibrosis where the profibrotic cytokine TGF-β plays a prominent role, e.g., UUO in mice. We first compared IL-15 and IL-15Rα mRNA expression in obstructed kidneys (UUO-kidneys) and contralateral non-obstructed kidneys (CTL-kidneys) on day-8 post-ureteral obstruction (Figure 1A). As expected, real-time reverse transcription PCR (RT-PCR) analysis, in UUO-kidneys compared with CTL-kidneys, demonstrated increased expression of TGF-β1 (*p* < 0.001, *n* = 7 per group) and TGFβI (transforming growth-factor beta-induced), which is a target gene of TGF-β1 (*p* < 0.01, *n* = 7 per group) that correlates with TGF-β1 activity in renal tissue [27]. Inversely, we observed a significant reduction at day 8 in the expression of both IL-15 and IL-15Rα in UUO-kidneys compared with CTL-kidneys (*p* < 0.05, *n* = 7 per group) (Figure 2).

### 2.3. IL-15/IL-15Rα Treatment Decreases Renal Fibrosis in the UUO Model

Given IL-15’s ability to maintain E-cadherin expression and attenuate collagen synthesis in tubular cells in vitro [26], we investigated whether the pharmacological delivery of IL-15 could thwart the endogenous IL-15 reduction and ameliorate TIF in an UUO model. First, 24 h before UUO experimental procedure and every other day thereafter, mice were intraperitoneally treated with IL-15 (2.5 µg) alone or with a mixture of IL-15 (2.5 µg) and soluble IL-15Rα-Fc (IL-15Rα; 15 µg). As markers of IL-15 treatments efficacy in vivo, we verified that the injection of the soluble IL-15/IL-15Rα complex, which greatly enhances IL-15 half-life and bioavailability in vivo [28,29], caused a marked increase in size spleens, in total numbers of CD8+ cells and NK cells and in pro-inflammatory cytokines expression (IFN-γ and RANTES) in spleen by day 8 in comparison to IL-15 alone (Figure S1).

**Figure 3:**
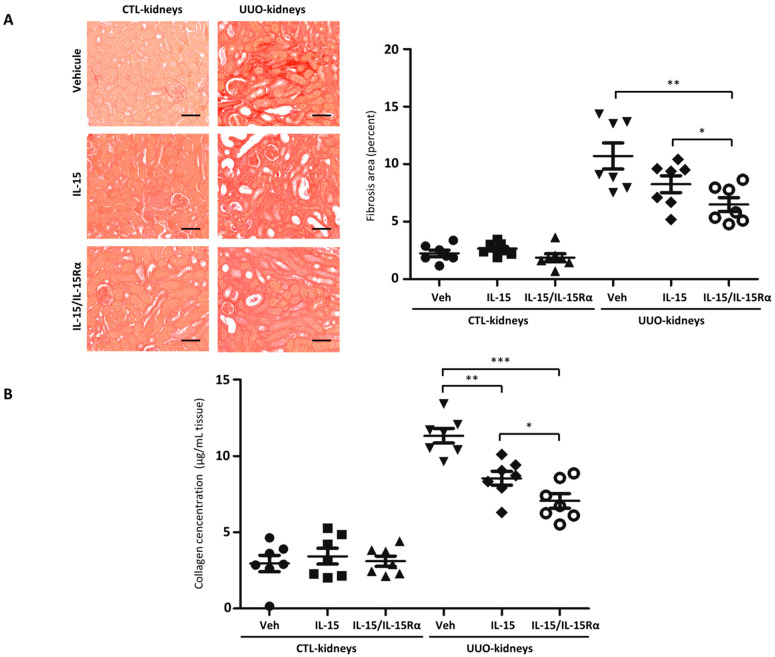
Pharmacological delivery of IL-15 and IL-15/IL-15Rα significantly reduces UUO-induced collagen deposition in fibrotic kidneys. (**A**) Left, Representative images of Sirius Red staining of kidney sections. Original magnification ×10. Scale bars, 50 μm. Right, Bar charts represent the quantification by histomorphometry of the positive area in renal cortex from vehicle (Veh), IL-15, and IL-15/IL-15Rα-treated mice after 8 days of UUO. Values are means ± SEM from seven mice by group. (**B**) Collagen content by hydroxyproline assay in IL-15 and IL-15/IL-15Rα-treated CTL and UUO kidneys (* *p* < 0.05, ** *p* < 0.01, *** *p* < 0.001, *n* = 7).

Then, we investigated the effect of both IL-15 treatments on the development of TIF in the UUO model. We quantified renal fibrosis using a histomorphometric analysis quantifying collagen content within an entire kidney cortex cross-section with Sirius Red (SR). As expected, a significant increase in Sirius Red staining of newly deposited collagen fibrils was observed in 8 d-post UUO-kidneys compared with CTL-kidneys (*p* < 0.001). IL-15/IL-15Rα-treated UUO-kidneys presented a 22% (*p* < 0.05) and 40% reduction (*p* < 0.01, *n* = 7 per group) in collagen accumulation (6.48 ± 0.60% of fibrosis area) compared with IL-15-treated mice (8.3 ± 0.73% of fibrosis area) and vehicle-treated mice (10.71 ± 1.13% of fibrosis area), respectively (Figure 3A).

In accordance with SR staining, colorimetric Hyp quantification in kidney lysates of the same mice were significantly higher in the UUO-kidneys (11.33 ± 0.47 µg Hyp/mg tissue) in comparison to CTL-kidneys (2.95 ± 0.53 µg Hyp/mg tissue, *p* < 0.001, *n* = 21). In accordance with SR staining, significantly less Hyp content was measured in both obstructed IL-15-treated (8.54 ± 0.46 µg Hyp/mg tissue, *p* < 0.01, *n* = 7) and IL-15/IL-15Rα-treated UUO-kidneys (7.06 ± 0.48 µg Hyp/mg tissue, *p* < 0.001, *n* = 7) compared to vehicle-treated UUO-kidneys after 8 days (Figure 3B). Among the other ECM components, IHC stainings reveal that fibronectin deposition was also reduced in both IL-15- and IL-15/IL-15Rα-treated UUO-kidneys when compared to vehicle-treated UUO-kidneys (Figure S2).

### 2.4. The IL-15/IL-15Rα Antifibrotic Effect in UUO Does Not Impact the Number of Myofibroblasts Accumulation

**Figure 4:**
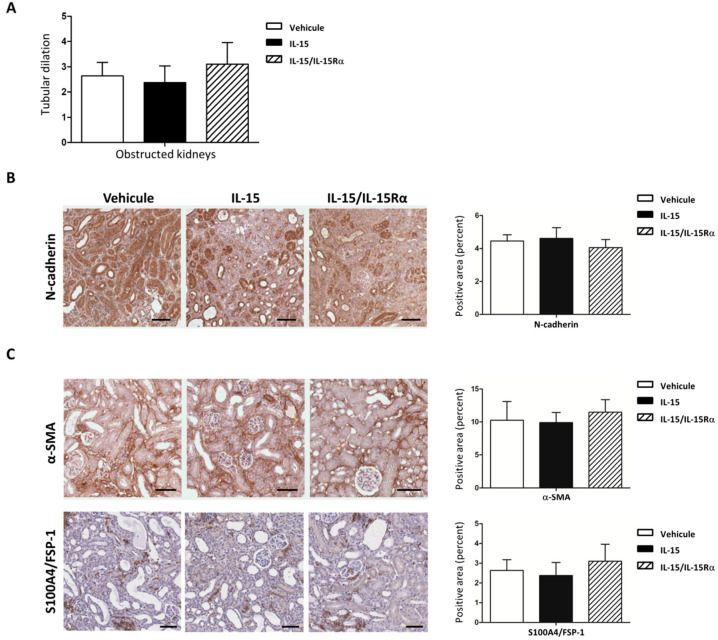
Neither IL-15 nor IL-15/IL-15Rα treatments modulated mesenchymal markers in 8 d-post obstructed kidneys. (**A**) Tubular dilation was quantified by two different experimenters as follows in a double-blind manner; score 0: 0% of dilated tubules, score 1: 0–25%, score 2: 25–50%, score 3: 50–75%, score 4: 75–100%. Data are means ± SEM. (**B**,**C**) Both IL-15 and IL-15/IL-15Rα treatments did not modify the mesenchymal markers expression in the renal cortex during UUO. Left, Representative IHC staining for *n*-cadherin, α-SMA, and S100A4/FSP-1 in the renal cortex of vehicle, IL-15, and IL-15/IL-15Rα-treated mice. Scale bars, 50 μm. Right, Bar charts represent quantification of the positive area by histomorphometry. Values are means ± SEM from seven mice by group.

In order to assess how IL-15 and IL-15/IL-15Rα reduce TIF during UUO, we first analyzed the tubular compartment, since we previously reported that IL-15 can directly counteract TGF-β1-induced EMT and excess matrix deposition in primary cultures of human renal proximal tubule epithelial cells [25,26]. Dilatation of collecting ducts was evident following 8 days of UUO in the obstructed kidney. However, no major difference in tubular dilation is observed during both IL-15 treatments compared with vehicle-treated mice (Figure 4A).

Similarly, we found no difference in *n*-cadherin, a marker of tubule differentiation, suggesting that the difference in renal fibrosis is not accompanied by preserved tubular structures (Figure 4B). RT-qPCR analysis demonstrated that the increase of TGFβI mRNA expression in UUO-kidneys was not modified by IL-15 or IL-15/IL-15Rα treatments (Figure 4A). We next analyzed the expression of mesenchymal markers α-SMA, S100A4/FSP-1 in the kidney cortex, which reflects myofibroblast number, although specific markers are lacking (Figure 4C). Semi-quantitative analysis showed that neither α-SMA nor S100A4/FSP-1 immunostaining was significantly different after both IL-15 treatments. These data suggest that the number of myofibroblasts expressing these markers was not modified by IL-15 in vivo.

### 2.5. The Role of IL-15 in Reducing Renal Fibrosis Involves a Direct Action on Renal Myofibroblasts

**Figure 5:**
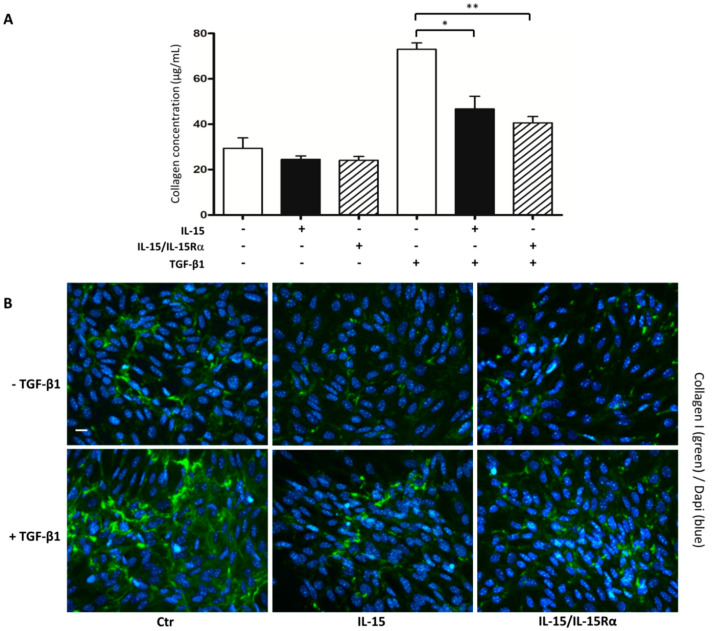
Both IL-15 treatments attenuated collagen synthesis and secretion induced by TGF-β1 stimulation in myofibroblasts. Myofibroblasts were treated for 48 h with TGF-β1 alone (2.5 ng/mL), IL-15 alone (2.5 ng/mL), or IL-15/IL-15Rα (2.5 ng/mL IL-15 + 15 ng/mL IL-15Rα-Fc). (**A**) The amount of collagen in 48 h treated myofibroblasts supernatants was quantified using the commercially Sirius Red collagen detection kit. Data are means ± SEM (* *p* < 0.05, ** *p* < 0.01, *n* = 3). (**B**) Immunofluorescent staining of collagen I expression (green) in 48 h treated myofibroblast cultures. Nuclei were stained with DAPI (blue). Representative images from three independent experiments are shown. Scale bars, 20 μm.

Despite reducing collagen and fibronectin accumulation, IL-15 had no effect on UUO-induced myofibroblast accumulation in the renal cortex. These data suggest that IL-15 could work downstream of myofibroblast activation to block ECM production. To evaluate this hypothesis, we used a primary cell culture of renal myofibroblasts to investigate whether IL-15 treatments could inhibit collagen synthesis in TGF-β1-stimulated myofibroblasts in vitro. Collagen secretion was quantified using the Sirius Red (SR) Total Collagen Detection Kit (Figure 5A). Thus, TGF-β1 treatment induced within 48 h a three-fold increase of the total soluble collagen levels in myofibroblast supernatants, while both IL-15 treatments partially inhibited TGF-β1 action (IL-15, *p* < 0.05; IL-15/IL-15Rα ** *p* < 0.01, *n* = 3) with the same efficiency. It is interesting to note that although not statistically, both IL-15 treatments showed also a tendency to decrease the baseline level of collagen secretion in untreated myofibroblast supernatants.

**Figure 6:**
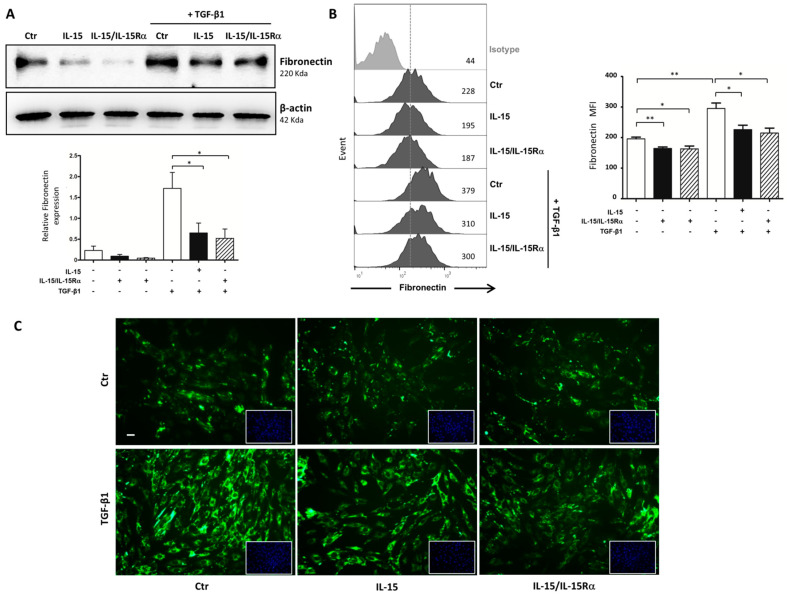
Both IL-15 attenuated fibronectin synthesis and secretion induced by TGF-β1 stimulation in myofibroblasts. (**A**) Western blot analysis of fibronectin expression after 48 h of TGF-β1 stimulation (2.5 ng/mL) in the presence or absence of IL-15 alone (2.5 ng/mL) or IL-15/IL-15Rα (2.5 ng/mL IL-15 + 15 ng/mL IL-15Rα-Fc). Upper, Representative Western blots of one experiment are shown. Lower, Bar chart represents fibronectin expression normalized to β-actin (* *p* < 0.05, *n* = 3). (**B**) Intracellular expression of fibronectin was assessed by flow cytometry in 48 h-treated myofibroblasts. Left, Representative staining of one experiment is shown. The dark-colored histograms correspond to cells incubated with an anti-fibronectin antibody, and gray histograms correspond to cells incubated with the isotype-matched control antibody. Mean fluorescence intensity values are shown at the right of each histogram. Right, Graphs represent the relative expression of fibronectin in four independent experiments. Data are shown as means ± SEM (* *p* < 0.05, ** *p* < 0.01, *n* = 4). (**C**) Immunofluorescent staining of fibronectin expression (green) in 48 h-treated myofibroblasts. Nuclei staining with DAPI (blue) was inserted at the bottom right of each image. Scale bars, 20 μm. Data are representative of three independent experiments.

Moreover, immunofluorescent studies (Figure 5B) confirmed that both IL-15 treatments markedly suppressed the strong increase of collagen I deposition in 48 h TGF-β1-treated myofibroblast cell cultures. Subsequently, we examined the prospective efficacy of IL-15 in inhibiting other ECM components, such as fibronectin. As expected, Western blot results (Figure 6A) and intracellular staining in flow cytometry (Figure 6B) revealed higher levels of fibronectin in 48 h TGF-β-treated myofibroblast supernatants. Interestingly, IL-15 treatments significantly reduced both basal and TGF-β1-increased fibronectin expression levels in the cell supernatants.

We further confirmed this inhibitory action of IL-15 on fibronectin downregulation by performing an immunofluorescence analysis which showed reduced fibronectin expression in IL-15-treated groups compared with TGF-β1 treatment (Figure 6C).

### 2.6. IL-15 Decreases Both Macrophage Infiltration and MCP-1 Expression in Obstructed Kidneys

**Figure 7:**
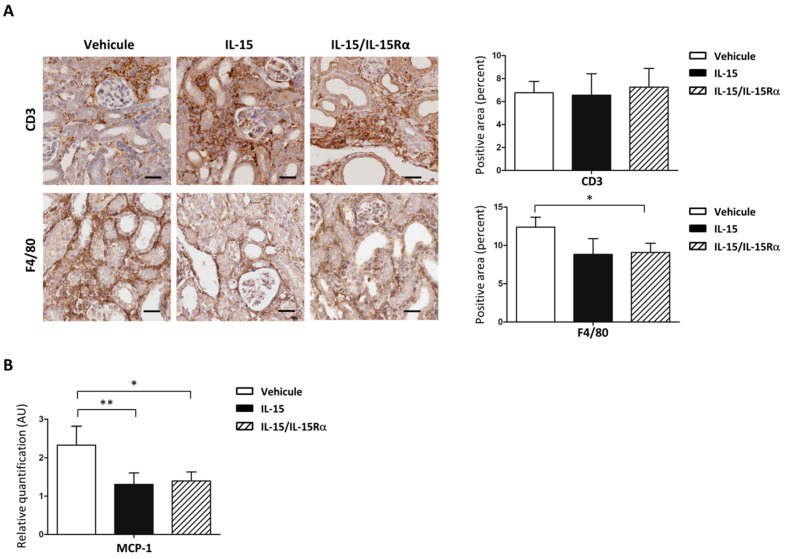
IL-15 decreases both macrophage infiltration and MCP-1 expression in 8 d-post obstructed kidneys. (**A**) Immunohistochemistry of CD3 and F4/80 positive cells in 8 d-post obstructed kidney cortex. CD3 lymphocyte infiltration in the kidney cortex was not modified by IL-15 (solid bars) and IL-15/IL-15Rα (hatched bars) treatments. On the other hand, both IL-15 treatments showed reduced infiltration of macrophages (F4/80+) during UUO. Left, Representative IHC staining of one experiment are shown. Scale bars, 25 μm. Right, Bar charts represent morphometric quantification. * *p* < 0.05 comparing vehicle (Veh, open bars) vs. IL-15/IL-15Rα (hatched bars)-treated mice, *n* = 7 per group. (**B**) RT-qPCR analysis of MCP-1 mRNA in kidney cortex normalized to Gus B mRNA. AU, arbitrary units. Values are means ± SEM from seven mice by group. ** *p* < 0.01 and * *p* < 0.05 comparing IL-15- or IL-15/IL-15Rα-treated mice to vehicle-treated mice.

Inflammation is a key process during UUO and triggers the first steps of renal fibrogenesis [30]. Since IL-15 or IL-15/IL-15Rα treatments restrained UUO-fibrosis, we performed a semi-quantitative analysis on the cells infiltrating the kidney cortex during UUO by immunostaining using T lymphocytes (CD3) and macrophages (F4/80) markers (Figure 7A). Then, 8 days after UUO, we confirmed a significant increase in both CD3 and F4/80 staining in the kidney, since CD3 and F4/80 staining are almost negative in CTL-kidneys (data not shown). IL-15 and IL-15/IL-15Rα treatments did not result in a significant reduction in CD3+ T cell infiltration compared to vehicle-treated UUO-kidneys. By contrast, F4/80+ cell infiltration was significantly decreased during UUO after IL-15/IL-15Rα treatment (IL-15-treated mice, *p* = 0.055; IL-15/IL-15Rα-treated mice, *p* < 0.05, *n* = 7 per group).

Monocyte chemoattractant protein 1 (MCP-1) is produced by a range of cell types in the kidney, including infiltrating leucocytes and intrinsic kidney cells, such as myofibroblasts. MCP-1 is one of the key chemokines involved in the migration of monocytes/macrophages to sites of active inflammation. Based on the ability of IL-15 to counter inflammation by inhibiting the induction of MCP-1 in a nephritis mouse model [23], we assessed by RT-qPCR MCP-1 levels in a UUO model (Figure 7B). An increase of MCP-1 mRNA expression in UUO-kidneys in comparison to CTL-kidneys was significantly decreased after IL-15 and IL-15/IL-15Rα treatments (*p* < 0.05, *n* = 7), suggesting that IL-15 could reduce macrophage infiltration in UUO kidneys through MCP-1 modulation.

### 2.7. IL-15 Inhibits TGF-β1 Induced MCP-1 Production by Myofibroblasts

**Figure 8:**
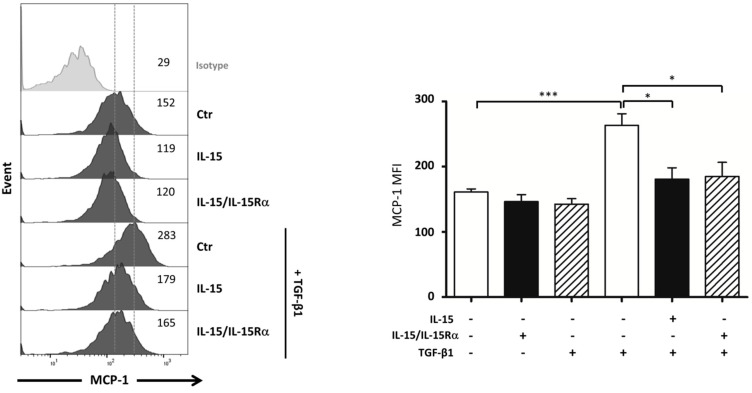
TGF-β1-increased MCP-1 expression in myofibroblasts is inhibited by both IL-15 treatments. Intracellular expression of MCP-1 was analyzed by flow cytometry in 48 h-treated myofibroblasts with TGF-β1 (2.5 ng/mL). Left, Representative staining of one experiment is shown. The dark-colored histograms correspond to cells incubated with an anti-MCP-1 antibody, and gray histograms correspond to cells incubated with the isotype-matched control antibody. Mean fluorescence intensity values are shown at the right of each histogram. Right, Graphs represent the relative expression of MCP-1 in four independent experiments. Data are shown as means ± SEM (* *p* < 0.05, *** *p* < 0.001, *n* = 4).

Subsequently, we investigated whether IL-15 could inhibit macrophage infiltration analyzing its effect on MCP-1 synthesis by myofibroblasts. In Figure 8, flow cytometry results reveal that TGF-β1 treatment strongly increases within 48 h of intracellular expression of MCP-1 in primary myofibroblast cultures. While IL-15 and IL-15/IL-15Rα treatments have no significant action on the baseline level of MCP-1, both IL-15 treatments markedly suppressed, with the same efficiency, TGF-β-induced MCP-1 expression (*p* < 0.05, *n* = 4). Thus, our results support the notion that the renoprotective effect of IL-15 observed in the UUO model could be related in part to its ability to counteract inflammation by inhibiting MCP-1 induction, as published in nephritis mouse models [23].

## 3. Discussion

Although renal fibrogenesis involves various pathways and cell types, myofibroblasts are believed to be major drivers of fibrotic disease progression through the production of excessive ECM in response to signals from damaged epithelial and inflammatory cells [31]. Among the mechanisms that have been studied to inhibit fibroproliferative events, strategies to target myofibroblast differentiation, activation, and proliferation emerge with high potential to limit ECM proteins accumulation in renal diseases [32]. Among the fibrotic factors, preclinical data suggest that TGF-β, which is responsible for myofibroblast differentiation and activation, is arguably the most potent profibrotic growth factor in kidney injury. However, recent clinical trials targeting TGF-β per se have been disappointing [10,11] due to its paradoxical functions in renal inflammation and fibrosis [33,34]. Further advances revealed promising TGF-β’s downstream targets or modulators of signaling (e.g., Smad7, Smad3-dependent noncoding RNA) to therapeutically target CKD [35,36,37]; however, none has emerged so far in the clinic. The innate pleiotropic cytokine IL-15, which is produced in the kidney, mainly by fibroblasts, monocytes, and epithelial cells, carries out activities opposite those of TGF-β. Therefore, IL-15 may bear an interesting but so far underestimated role in preventing renal fibrogenesis. In addition to its role as a survival factor for renal epithelium [24], we previously reported that IL-15 can directly counteract TGF-β1-induced epithelial–mesenchymal transition (EMT) and excess matrix deposition in primary cultures of human renal proximal tubule epithelial cells [25,26]. Interestingly, a growing body of evidence supports a negative correlation between IL-15 and TGF-β expression during renal fibrogenesis. Indeed, IL-15 protein expression is drastically decreased in chronic inflammatory nephropathies evolving toward fibrosis, such as acute interstitial nephritis (AIN), IgA nephropathies, and diabetic nephropathy [26]. While IL-15 transcripts are down-regulated in biopsies of transplanted patients with renal dysfunction in comparison to transplant patients from well-functioning transplants [26], our results confirmed that IL-15 expression is also decreased in nephrectomized allografts with terminal dysfunction compared with normally functioning kidneys (Figure 1). Furthermore, similar to reports in renal injury models [23,24], we found that both IL-15 and IL-15Rα expressions were decreased in UUO-kidneys compared with CTL-kidneys (Figure 2). Taken together, these data suggest that IL-15 reduction, as observed for the antifibrotic factors, such as BMP-7 [13,38] and HGF [39], may be a critical event in renal fibrogenesis and in acute and chronic kidney diseases. Importantly, a decline in the intrarenal expression of IL-15Rα is also detrimental to the biological activity of IL-15, since this chain IL-15Rα, that specifically binds IL-15 with a high affinity, is involved in complex mechanisms through several types of receptors and transmembrane and soluble forms of the cytokine [28,29,40,41,42].

Given the emerging evidence that IL-15 reduction may be a critical event in renal fibrogenesis and the fact that IL-15 exerts renal anti-apoptotic and antifibrotic activities in vitro, we sought to confirm the role of IL-15 in preventing renal fibrogenesis. Thus, we investigated whether pharmacological IL-15 delivery could thwart endogenous IL-15 reduction and restrain fibrosis in a mouse model of UUO, which is a fast and reproducible model of tubulointerstitial fibrosis (TIF) involving inflammation. These investigations provide the first in vivo evidence that IL-15 treatment can restrain renal fibrogenesis, as reflected by reduced total collagen content observed with SR staining and Hyp content measurement in both IL-15- and IL-15/IL-15Rα-treated UUO-kidneys when compared to vehicle-UUO-kidneys (Figure 3). In particular, IL-15 treatments reduced collagen 3 deposition in 8 d-post obstructed kidney tissues (Figure S3). Moreover, IHC stainings reveal that fibronectin deposition was also reduced in both IL-15- and IL-15/IL-15Rα-treated UUO-kidneys when compared to vehicle-treated UUO-kidneys (Figure S2), demonstrating that IL-15 treatments reduce drastically the interstitial production of ECM proteins, thereby preventing UUO-induced renal fibrosis. The higher efficiency of IL-15/IL-15Rα treatment compared to IL-15 alone in the 8-days UUO model (Figure 3 and Figure S1) is explained by the fact that the IL-15/IL-15Rα complex, with increased biological activity and IL-15 half-life, would represent the predominant form of the cytokine [20,28,29].

First, the antifibrotic effects of IL-15 in UUO-kidneys may not be related to TGF-β activity, since mRNA expressions of TGF-β1 and TGFβI, a target gene of TGF-β1 that correlates with TGF-β1 activity in renal tissue [27], were not modified by both IL-15 treatments (Figure 2). Moreover, we found no difference in the expression of mesenchymal markers expression (α-SMA, S100A4/FSP-1, *n*-cadherin (Figure 4), vimentin (data not shown)) by IHC in fibrotic kidneys from both vehicle- and IL-15-treated animals. In vivo, these markers may reflect tubular injury and myofibroblast number as well. In particular, α-SMA and S100A4/FSP-1 are two hallmark markers of the myofibroblast [43,44]. Taken together, these data suggest that the total pool of myofibroblasts in renal interstitium at 8 days of UUO was not modified by IL-15 treatments. Although IL-15 may inhibit TGF-β-induced EMT in primary renal epithelial culture cells [26], the lack of difference in terms of mesenchymal markers is not totally unexpected, considering that the EMT process is weakly involved in the generation of myofibroblasts in the UUO model. Indeed, a lineage tracing experiment in a UUO model reported that the total pool of myofibroblasts comes mainly from the proliferation of local resident fibroblasts (50%) and differentiation of bone marrow precursors (35%), while the endothelial to mesenchymal transition program (EndMT) and EMT represent only 10% and 5%, respectively [6]. Therefore, the fact that IL-15 abrogated fibrosis and ECM deposition despite an inability to alter myofibroblast activation suggests that IL-15 works downstream of myofibroblast activation to block ECM production. To test the hypothesis that IL-15 exerts a direct action on myofibroblasts, we used a primary cell cultures of myofibroblasts derived from explants of vehicle-treated UUO-kidneys. With the same efficiency, both IL-15 treatments inhibited collagen and fibronectin secretion by TGF-β1-treated myofibroblasts (Figure 5 and Figure 6). Interestingly, both IL-15 treatments showed also a tendency to decrease the baseline level of collagen and fibronectin secretion in untreated myofibroblast supernatants, suggesting that IL-15 could inhibit endogenous activated TGF-β in primary myofibroblast cultures. These results suggest that the renoprotective effect of IL-15 in UUO acts, at least in part, through a direct inhibition of ECM synthesis by myofibroblasts, which is the major effector cell during renal fibrogenesis. It is noteworthy that BMP-7 exerts similar antifibrotic activities in an UUO model, since the delivery of exogenous BMP-7 showed marked improvement of TIF by reducing TGF-β-induced ECM production without affecting the pool of myofibroblasts [13]. Collagen deposition during fibrogenesis is dependent not only on an increased synthesis of proteins but also on decreased collagen turnover [45,46]. It remains to be further investigated if the reduced collagen accumulation observed in IL-15-treated UUO-kidneys is also due to the enhanced degradation of ECM.

Inflammation is seen as an important component and driver of renal fibrogenesis, as in UUO, since renal fibrosis can be attenuated when macrophage and T-cell recruitment is reduced [47,48]. Indeed, in the UUO model, interstitial inflammation is seen as early as 3 days after obstruction with a predominance of monocyte/macrophages infiltration [49,50]. While pharmacological delivery of IL-15/IL-15Rα exhibits more potent immunostimulatory effects on NK and CD8+ T cells in the spleen than IL-15 alone (Figure S1), both IL-15 treatments failed to modify T lymphocyte infiltration within the kidney. Nevertheless, IL-15 and IL-15/IL-15Rα treatments reduced renal macrophage infiltration with the same efficiency (Figure 7A). This reduction of renal macrophage infiltration could be explained by a decreased expression of the chemotactic protein MCP-1 in UUO-kidneys after IL-15 and IL-15/IL-15Rα treatments (Figure 7B). This conclusion is supported by experiments showing that both IL-15 treatments can inhibit MCP-1 production in primary myofibroblast cultures treated with TGF-β (Figure 8). Our findings support those of Shinozaki et al. showing that the renoprotective effect of IL-15 observed in a nephritis mouse model has been related in part to its ability to counteract inflammation by inhibiting MCP-1 induction [23]. Indeed, MCP-1 is produced by a wide range of cell types in the kidney, including infiltrating leucocytes (e.g., macrophages) and intrinsic kidney cells (e.g., epithelial cells, fibroblasts) and is considered as a major chemoattractant chemokine for monocytes/macrophages [51,52]. Thus, MCP-1 expression is involved in the severity of various nephropathies [53,54], and its attenuation decreases kidney damage by reducing inflammation in different injury models [55,56]. In addition to its chemoattractive effect on monocyte/macrophages, the main TGF-β producers [57], MCP-1 itself could stimulate collagen synthesis and endogenous up-regulation of TGF-β expression in fibroblasts, leading to autocrine and/or juxtacrine stimulation of collagen gene expression [58].

Our current findings in UUO demonstrate that IL-15 could reduce ECM deposition through a direct action on myofibroblast collagen synthesis, but also, indirectly, by reducing inflammation, in particular macrophage infiltration, through the down-regulation of MCP-1 synthesis by myofibroblasts. However, it is likely that IL-15 may exert antifibrotic effects through other mechanisms in CKD fibrogenesis, as reported in other organs. Thus, while endogenous IL-15 deficiency induces profibrotic cytokines (e.g., TGF-β) and myofibroblast accumulation in allergic lung [17] and chronic pancreatitis [18] models, pharmacological IL-15 delivery ameliorates fibrosis by down-regulating TGF-β and by restricting the TGF-β1-induced myofibroblast differentiation. Mechanistically, the IL-15 antifibrotic role was associated with the induction of interferon-γ-responsive invariant natural killer T (iNKT) cells or T-helper cell type 17 suppressor RORγ+ Treg cells in chronic pancreatitis models [18] and in allergen lung fibrosis models [17] respectively. In addition, Wuttge et al. reported that IL-15 can counteract directly TGF-β1-induced myofibroblast differentiation of human fetal lung fibroblasts [22]. Moreover, the protective role of IL-15 in murine hepatic fibrosis has been linked to its ability, on the one hand, to preserve NK homeostasis and, on the other hand, to directly inhibit the trans-differentiation of hepatic stellate cells into myofibroblasts [19]. Further experiments are needed to better understand the complexities of action of IL-15 on both innate lymphoid cells and renal cellular components (e.g., epithelial cells, fibroblasts), especially in renal fibrosis. In a previous study, we reported that IL-15 can counteract TGF-β induced-EMT in primary tubular epithelial cells [26]. Thus, it is tempting to speculate that IL-15 antifibrotic actions on tubular epithelial cells may be involved in CKD, such as diabetic nephropathy, in which the EMT process has been more clearly associated with renal fibrogenesis [59,60]. Another limitation of our study is that UUO is a unilateral model of TIF without renal function as an end point. As CKD is defined as gradual reduction in kidney function, manifesting itself as a permanent reduction in glomerular filtration rate, further models of renal fibrosis are needed to assess whether inhibition of the progression of fibrosis by IL-15 treatment leads to an improvement in glomerular filtration rate and reduced proteinuria. Moreover, apart from tubules and myofibroblasts, Luque et al. suggested that IL-15 may exert a protective direct role in podocytes in the experimental anti-glomerular basement membrane glomerulonephritis (anti-GBM-GN) model. They found that the common gamma chain (γC) or the interleukin-2 receptor β subunit (IL-2Rβ) deletion aggravated the anti-GBM-GN. Additional in vitro data suggested a direct signaling of IL-15 acting through the γC in podocytes [61]. Further studies would be required to specifically address these questions.

Altogether, the intrarenal expression of IL-15 may represent a key functional modus operandi that limits renal fibrogenesis associated with CKD. As other endogenous antifibrotic factors, such as BMP-7 and HCG, the survival factor IL-15 could be a natural antagonist of TGF-β in human kidney allowing epithelial homeostasis. In this context, an increase in the expression of TGF-β observed in multiple nephropathies, which is associated with a decrease in the expression of IL-15, may be critical in promoting fibrogenesis and renal failure. In this study, we demonstrated the antifibrotic effect of IL-15 in an experimental model of renal fibrosis. We highlighted that IL-15 per se prevents renal fibrosis through a direct action on collagen synthesis on myofibroblasts as well as by reducing macrophage infiltration in UUO. Overall, our study demonstrates that IL-15 may be an important player in renal fibrosis and may be considered as a novel therapeutic agent in renal disease as a repurposing drug.

## 4. Materials and Methods

### 4.1. Human Kidney Specimens

We used graft obtained after transplantectomy for chronic allograft dysfunction of non-immune origin. Kidney donation followed the Declaration of Istanbul principles and the French Agence Nationale de la Biomedecine regulation. Informed written consent was given by the patients for the use of part of the biopsy for scientific purposes. All procedures and the use of tissues were performed in accordance with the Declaration of Helsinki principles. We also used renal kidneys from deceased donors that were deemed unsuitable for transplantation for anatomical reasons or due to excessive cold ischemia time. Our protocol was approved by the French Agence Nationale de la Biomedecine under the number PFS14-019.

### 4.2. Animals

C57BL/6 mice were purchased from Janvier Laboratory (Le Genest-Saint-Isle, France). All mice were housed under standardized conditions with a 12 h dark/light cycle and had unlimited access to food and water. All animal procedures were approved by the animal ethics advisory committee of Paris Sud University number 26 and was approved by the Ministère de l’Education Nationale, de l’Enseignement Supérieur et de la Recherche. Each mouse from the IL-15 group received 2.5 µg of recombinant human IL-15 (rhIL-15) (Miltenyi Biotec, GmbH, Bergisch Gladbach, Germany #130-095-765) in 200 µL of PBS. The IL-15/IL-15Rα group was treated with 2.5 µg of rhIL-15 precomplexed for 30 min at 37 °C with 15 µg of recombinant mouse (rm)IL-15Rα-Fc chimeric molecule (Bio-Techne Ltd., Lille, France, #551-MR) in 200 µL of PBS. Vehicle mice were treated with 200 µL PBS. All treatments were administered subcutaneously one day before obstruction and every other day.

### 4.3. Unilateral Ureteral Obstruction (UUO)

After giving general anesthesia (isoflurane 2%), the left ureter was exposed via a mid-abdominal incision. UUO was performed with double silk sutures by complete ligation of the left ureter. The contralateral kidney served as an intra-individual control. Animals were sacrificed on day 8 after treatment.

### 4.4. Cell Culture

Primary cultures of mouse kidney myofibroblasts were isolated from the explantation of 8-day post-ligation renal cortical tissue of C57/BL6 mice, as previously described [56,62]. Myofibroblasts were grown in DMEM with 4.5 g/L glucose and glutamine (Lonza, Walkersville, MD, USA), 25 mM HEPES pH 7.4 (Sigma-Aldrich, St. Quentin Fallavier, France), 100 µg/mL streptomycin, 100 U/mL penicillin (Pen/Strep), and 10% FCS (Linaris, LaborChemie, Vienna, Austria). Myofibroblasts were pretreated overnight with rhIL-15 (2.5 ng/mL) or the soluble IL-15/IL-15Rα complex composed of rhIL-15 (2.5 ng/mL) precomplexed for 30 min at 37 °C in PBS with 15 ng/mL of rmIL-15Rα-Fc chimeric molecule. Then, myofibroblasts were treated with 2.5 ng/mL of rmTGF-β1 (Bio-Techne Ltd., Lille, France, #7666-MB/CF) for 48 h.

### 4.5. Immunohistochemistry

Paraffin-embedded kidney tissues were deparaffinized, rehydrated, and treated by the appropriate antigen retrieval method (heat-induced epitope retrieval method using sodium citrate 10 mM, pH 6.0 in a pressure cooker, or enzymatic method using 20 µg/mL proteinase K at 37 °C for 15 min). They were stained with antibodies against α-SMA (Abcam, Cambridge, UK, ab5694), N-cadherin (Abcam, Cambridge, UK, ab12221), CD3 (DakoCytomation, Glostrup, Denmark, A0452), and F4/80 (Abcam, Cambridge, UK, ab6640), and the Envision kit was applied for 45 min at room temperature (DakoCytomation, Denmark) according to the manufacturer’s instructions. Staining was revealed by applying a DAB kit (DakoCytomation, Glostrup, Denmark) and counterstained with hematoxylin (Sigma-Aldrich, St. Quentin Fallavier, France). Positive staining was quantified using computer-based morphogenic analysis software (TRIBVN ICS Framework) in a blinded manner. The positive area in the cortex was measured for each specimen and expressed as a percentage of the total cortical kidney section.

### 4.6. Histopathological Analysis of Renal Fibrosis

Kidneys were fixed in 10% formalin and embedded in paraffin. Tissue sections (4 µm) were stained with picro-Sirius Red. Renal cortex fibrosis was quantified using the computer-based morphogenic analysis software (Calopic version 2.10.16, TRIBVN Healthcare, Châtillon, France) in a blinded manner. The total kidney cortex of an entire kidney sagittal cross-section was selected for quantification from each mouse, representing an approximate 7 mm^2^ surface area. The positive area was expressed as a percentage of the total cortical kidney section.

### 4.7. Quantification of Collagen Deposition in Obstructed Kidneys by Hydroxyproline Measurement

The colorimetric QuickZyme total collagen assay was used for the quantification of collagen deposition by hydroxyproline (Hyp) measurement (QuickZyme Biosciences, Leiden, The Netherlands, #QZBtotcol1). Aliquots of 10–25 mg of snap frozen mouse renal tissues were first hydrolyzed in 6 mol/L HCl for 20 h at 95 °C in a thermoblock. The lysates were diluted to 4 mol/L HCl and then added in triplicates to a 96-well plate. Then, assay buffer and detection reagent were added, and the plate was incubated for 60 min at 60 °C and read at 570 nm on a microplate reader (Bio-Rad Laboratories, Hercules, CA, USA). A calibration curve was constructed using Hyp in the range of 12.5–300 μM and readouts of the collagen assay were normalized to tissue weight.

### 4.8. Quantification of Collagen Secretion by Myofibroblasts

The amount of collagen in the cell supernatant of 48 h-treated cells was quantified using the commercially Sirius Red collagen detection kit (Chondrex, Inc., Redmond, WA, USA, #9062) as per the manufacturer’s instructions. Briefly, collagen was first concentrated using the Concentrating Solution (Chondrex, Inc., Redmond, WA, USA, #90626) at 4 °C for 24 h. After dissolving the pellet in acetic acid, samples were incubated with Sirius Red solutions for 30 min at room temperature and eluted using an extraction buffer. The absorbance of the extracted solution was read at 540 nm by microplate reader. A calibration curve was constructed using bovine collagen-I in the range of 8–250 μg/mL.

### 4.9. Real-Time Quantitative PCR

RNA was extracted from tissues or cells using a QIAGEN RNeasy Mini kit, according to the manufacturer’s protocol. cDNA was synthesized using a Revert Aid H minus First Strand DNA Synthesis kit (Fermentas, Villebon-sur-Yvette, France) and amplified by PCR with a LightCycler 480 (Roche Diagnostics, Meylan, France) using SYBR Green (Fast Start DNA Master SYBRGreen I, Roche Applied Science, Roche Diagnostics). Specific primers were used (Table S1) under the following conditions: 95 °C for 5 min, 45 cycles at 95 °C for 15 s and at 60 °C for 15 s, then 72 °C for 15 s.

### 4.10. Total Lysate from Kidneys and Cells

The kidney cortex was homogenized in lysis buffer (50 mM Tris-HCl, 150 mM NaCl, 1% NP-40, 10% glycerol, 1 mM orthovanadate, 25 mM β-glycerophosphate, 1 mM PMSF, and a complete protease inhibitor cocktail (Roche Diagnostics, Meylan, France), pH 7.4); then, supernatants were collected. Cell pellets were lysed in 1% Triton X-100, 20 mM Tris-HCl (pH 7.4), 137 mM NaCl, 2 mM EDTA, 2 mM sodium pyrophosphate, and 10% glycerol in the presence of phosphatase inhibitors (25 mM β-glycerophosphate and 1 mM sodium orthovanadate) and complete protease inhibitor cocktail tablets (Roche Diagnostics, Meylan, France).

### 4.11. Western Blot Analysis

Proteins were quantified by BCA protein assay kit (Thermo Fisher Scientific, Illkirch, France), and 35 μg of protein were loaded in Laemmli buffer and were resolved by SDS-PAGE. Proteins were transferred onto a polyvinylidene difluoride membrane in an electrophoretic transfer cell system (Thermo Fisher Scientific, Illkirch, France) for 5–7 min. Membranes were immediately blocked with solution containing 5% nonfat dry milk or BSA in 0.1% TBS-Tween 20 (TBST). Blots were subsequently probed overnight with antibodies against IL-15 (SantaCruz, Dallas, TX, USA, sc-1296), α-SMA (Abcam, Cambridge, UK, ab5694), TGF-β (SantaCruz, Dallas, TX, USA, sc-52893), and β-actin-HRP (SantaCruz, Dallas, TX, USA, sc-47778). Appropriate HRP-conjugated secondary antibodies were used (1:5000, Jackson Immunoresearch).

### 4.12. Flow Cytometric Analysis

Primary myofibroblast cultures were treated in vitro with IL-15, IL-15/IL-15Rα complex, and/or TGF-β for 48 h in the presence of monensin (2.0 mM; Thermo Fisher Scientific, Illkirch, France, #00-4505) for the last 18 h. Cells were detached with Accutase (Sigma-Aldrich, St. Quentin Fallavier, France, A6964), and intracellular expression of MCP-1 and fibronectin was assessed by flow cytometry as previously described [63]. Briefly, after fixation and permeabilization using cytofix/cytoperm solution (BD Biosciences, Franklin Lakes, NY, USA), cells were stained with antibodies against MCP-1 (Thermo Fisher Scientific, Illkirch, France, #MA5-17040) or fibronectin (Abcam, Cambridge, UK, Ab2413) for 45 min in the dark at 4 °C, washed with 1× Perm/Wash solution, and labeled with a phycoerythrin (PE)-conjugated secondary antibody for 45 min in the dark at 4 °C. After labeling, cells were washed once and analyzed in an LSR Fortessa™ cell analyzer ( BD Biosciences, Franklin Lakes, NY, USA), and the data were analyzed using FlowJo software (Tree Star Inc., Ashland, OR, USA). The experiment was repeated at least three times.

### 4.13. Immunofluorescence Staining

Cells were fixed with 4% (*w*/*v*) paraformaldehyde in PBS and permeabilized by incubation for 1 min with 0.5% Triton X-100. Then, cells were incubated with blocking solution (3% BSA in PBS) and incubated overnight at 4 °C with primary antibodies against collagen I (Abcam, Cambridge, UK, ab34710) and fibronectin (Abcam, Cambridge, UK, Ab2413). Fluorescent immunostaining was revealed with Alexa Fluor 488-conjugated secondary antibodies (Jackson Immunoresearch Laboratories, West Grove, PA, USA). 4′,6-Diamidino-2-Phenylindole, Dihydrochloride (DAPI, Thermo Fisher Scientific, D1306) was used to stain cell nuclei. The confocal images were captured by a confocal LEICA SP5-AOBS microscope with a 63X/1.4 NA oil-immersion objective.

### 4.14. Statistical Analyzes

The data were analyzed by GraphPad Prism 8.0.1. Statistically significant differences were determined using the Mann–Whitney U-test or *t*-test, as appropriate. Data were considered statistically significant when the *p*-value was <0.05. Error bars represent standard error of the mean (±SEM) of independent experiments.

## Data Availability

The data presented in this study are available upon request from the corresponding author.

## Supplementary Materials

The following are available online at https://www.mdpi.com/article/10.3390/ijms222111698/s1, Figure S1: IL-15/IL-15Rα complex induces a marked increase in total numbers of CD8+ cells and NK cells in spleen, Figure S2: Both IL-15 and IL-15/IL-15Rα treatments reduced fibronectin deposition in 8d-post obstructed kidneys (UUO-kidneys), Figure S3: Both IL-15 and IL-15/IL-15Rα treatments reduced collagen 3 deposition in 8d-post obstructed kidneys (UUO-kidneys), Table S1: Sequences of primers used for real-time quantitative PCR amplification.
