# Supplementary material for: IL-15 Prevents Renal Fibrosis by Inhibiting Collagen Synthesis: A New Pathway in Chronic Kidney Disease?
Source: Int J Mol Sci. 2021 Oct 28;22(21):11698. doi: 10.3390/ijms222111698 (PMC8583733; doi:10.3390/ijms222111698)
Supplement: Supplementary file 1 [file ijms-22-11698-s001.zip › Table S1 editable.pptx]

## Slide 1
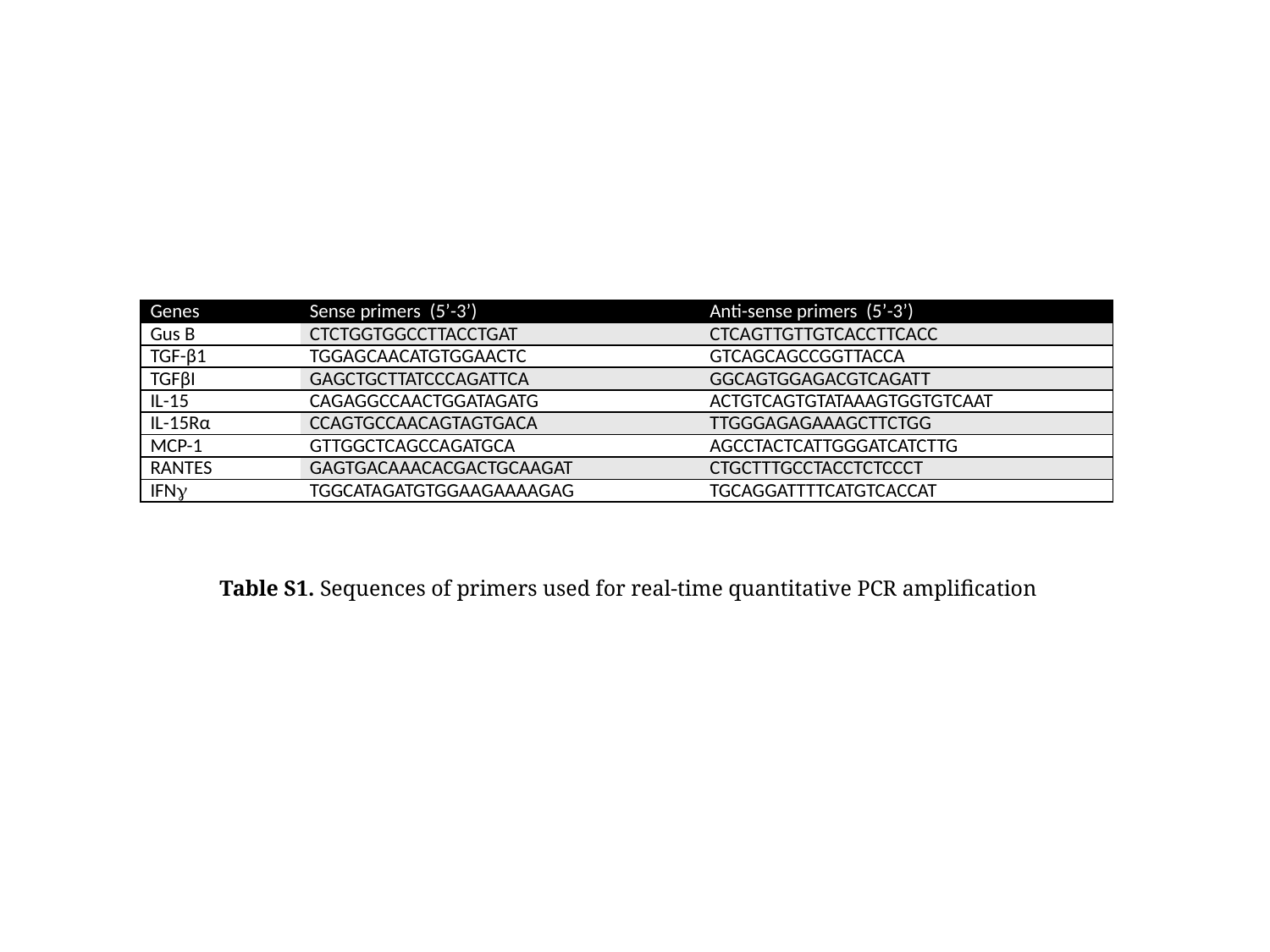

| Genes | Sense primers (5’-3’) | Anti-sense primers (5’-3’) |
| --- | --- | --- |
| Gus B | CTCTGGTGGCCTTACCTGAT | CTCAGTTGTTGTCACCTTCACC |
| TGF-β1 | TGGAGCAACATGTGGAACTC | GTCAGCAGCCGGTTACCA |
| TGFβI | GAGCTGCTTATCCCAGATTCA | GGCAGTGGAGACGTCAGATT |
| IL-15 | cagaggccaactggatagatg | actgtcagtgtataaagtggtgtcaat |
| IL-15Rα | ccagtgccaacagtagtgaca | ttgggagagaaagcttctgg |
| MCP-1 | GTTGGCTCAGCCAGATGCA | AGCCTACTCATTGGGATCATCTTG |
| RANTES | GAGTGACAAACACGACTGCAAGAT | CTGCTTTGCCTACCTCTCCCT |
| IFNγ | TGGCATAGATGTGGAAGAAAAGAG | TGCAGGATTTTCATGTCACCAT |
Table S1. Sequences of primers used for real-time quantitative PCR amplification
