# Supplementary figures and images for: IL-15 Prevents Renal Fibrosis by Inhibiting Collagen Synthesis: A New Pathway in Chronic Kidney Disease?
Source: Int J Mol Sci. 2021 Oct 28;22(21):11698. doi: 10.3390/ijms222111698 (PMC8583733; doi:10.3390/ijms222111698)

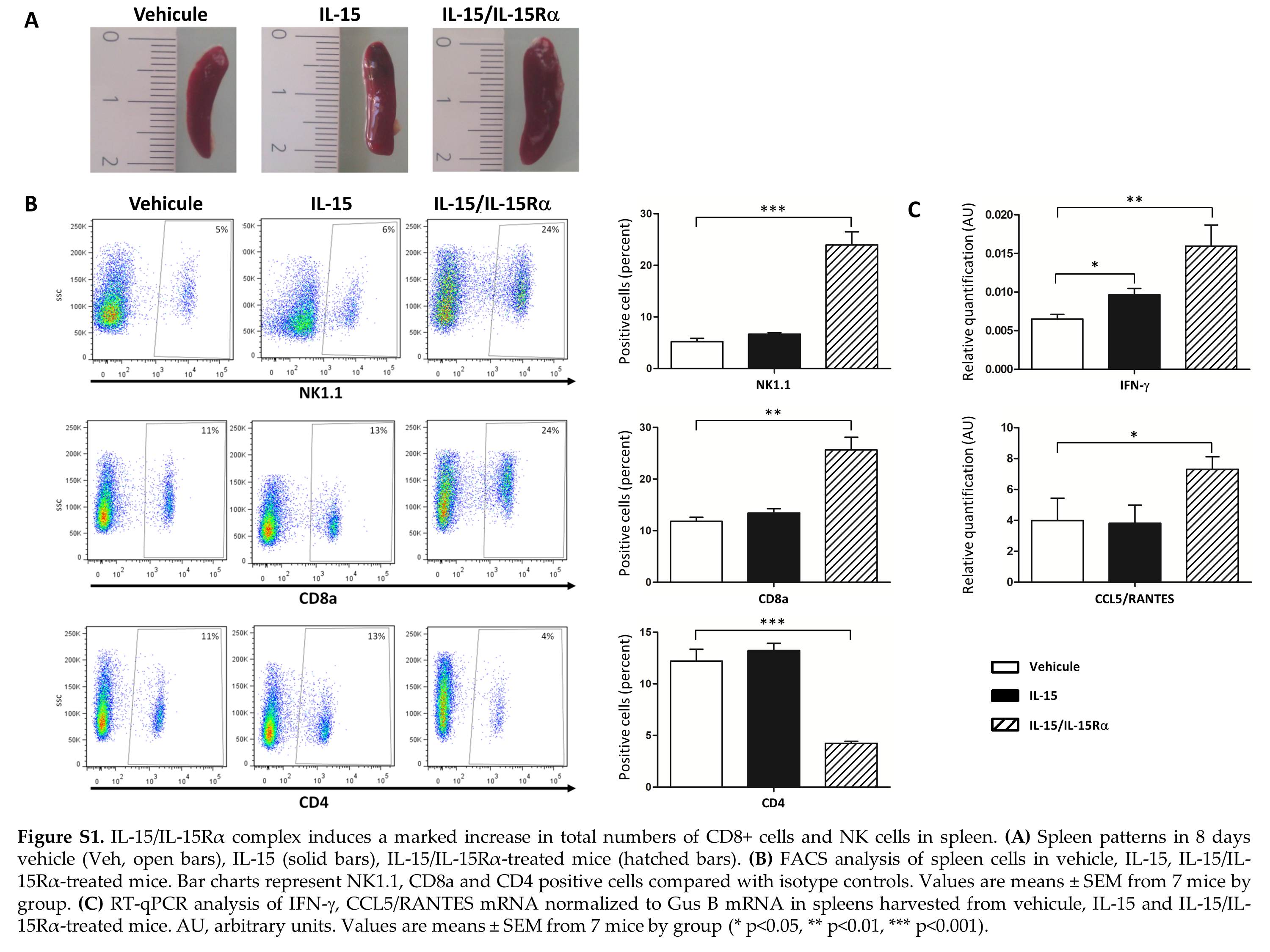

Supplement: Supplementary file 1 [file ijms-22-11698-s001.zip › Figure S1.TIF]

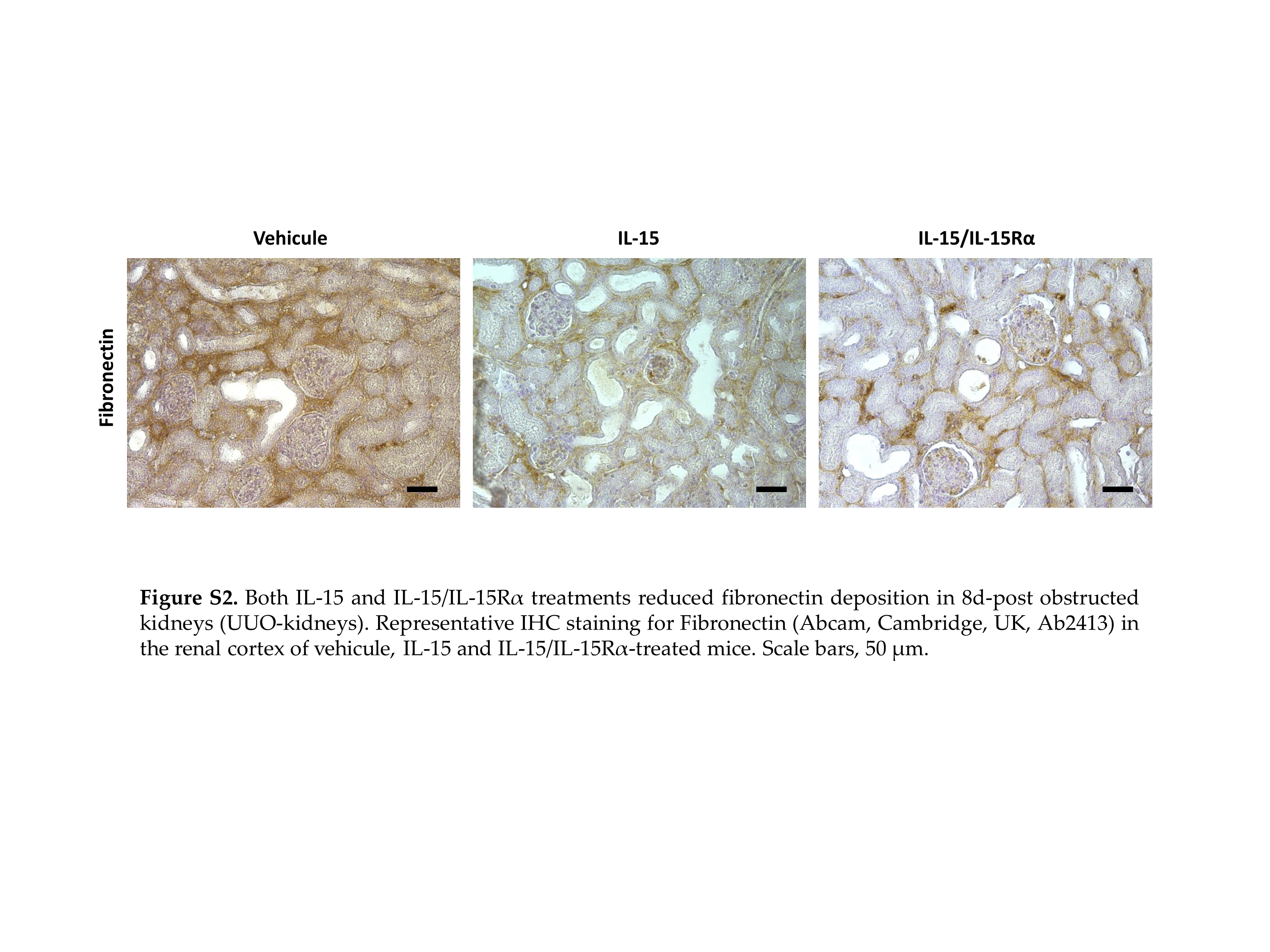

Supplement: Supplementary file 1 [file ijms-22-11698-s001.zip › Figure S2.TIF]

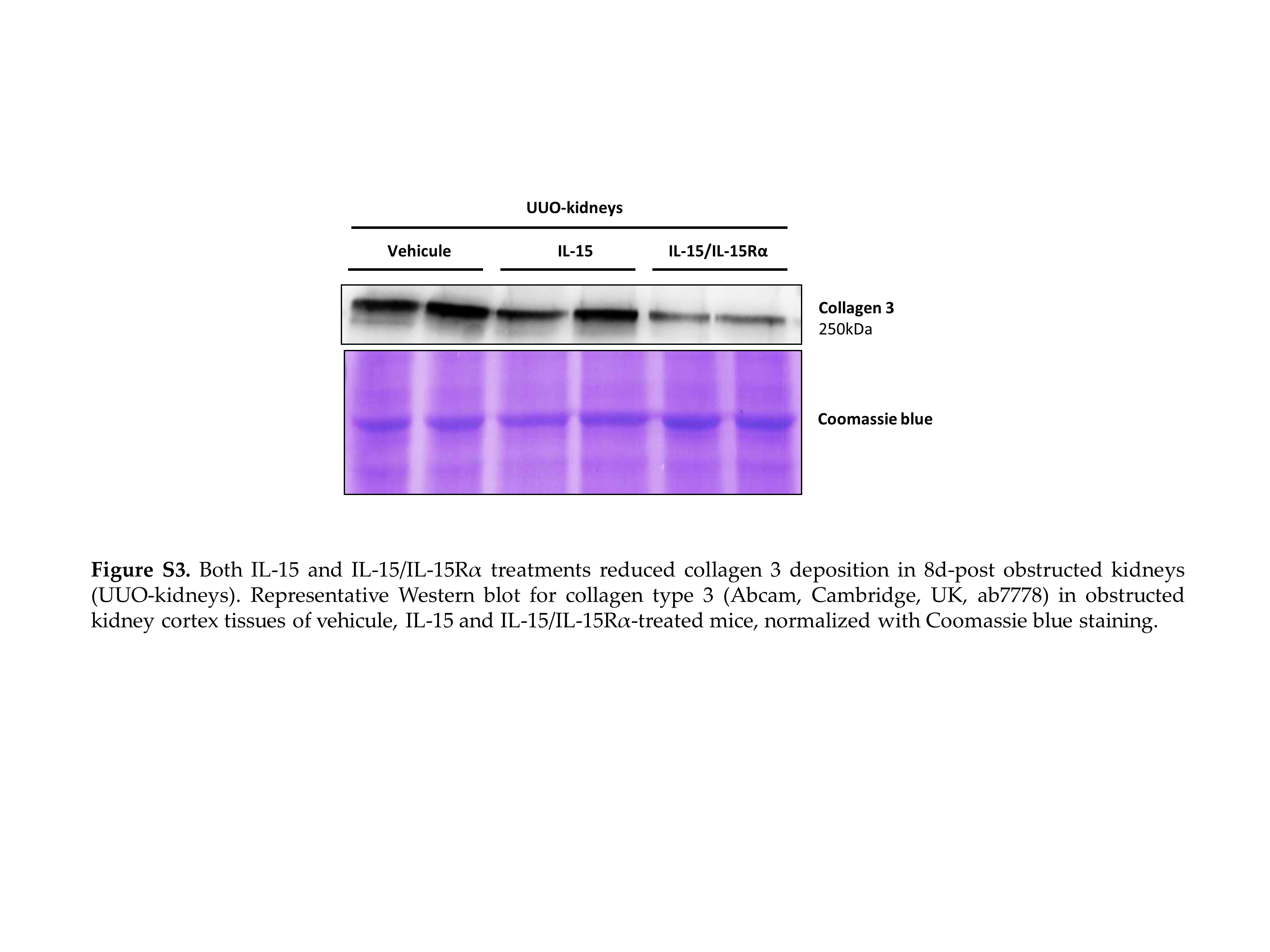

Supplement: Supplementary file 1 [file ijms-22-11698-s001.zip › Figure S3.TIF]
